# Supplementary material for: Interaction of Vinyl-Type Carbocations, C3H5+ and C4H7+ with Molecules of Water, Alcohols, and Acetone
Source: Molecules. 2023 Jan 23;28(3):1146. doi: 10.3390/molecules28031146 (PMC9921539; doi:10.3390/molecules28031146)
Supplement: Supplementary file 1 [file molecules-28-01146-s001.zip › molecules-2112650-supplementary.pdf]

molecules

MDPI

Supplementary Materials

Interaction of Vinyl-Type Carbocations, $C_3H_5^+$ and $C_4H_7^+$ with Molecules of Water, Alcohols, and Acetone

Evgenii S. Stoyanov *, Irina Yu Bagryanskaya and Irina V. Stoyanova

N.N. Vorozhtsov Institute of Organic Chemistry, Siberian Branch of Russian Academy of Sciences, 630090 Novosibirsk, Russia
* Correspondence: evgenii@nioch.nsc.ru

IR spectral and X-ray structural data

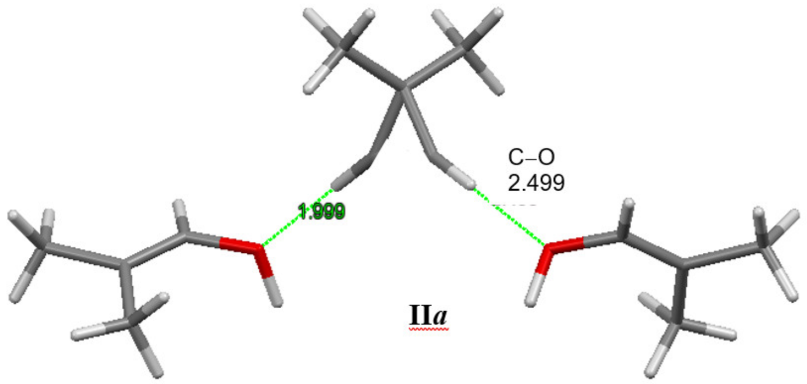

Figure S1. Two locations of the $C_4H_7^+ \cdot C_4H_7OH$ adduct in the crystal lattice of its salt with the $\{Cl_{11}^-\}$ anion (not shown). The structure of protonated ether $C_4H_7–O^+(H) –C_4H_8Cl$ (as a salt of the $\{Cl_{11}^-\}$ anion) was determined by X-ray diffraction analysis; the C=C bond length in the $(H_3C)_2C=C^+(H)$ molecular fragment is 1. 278 Å, and the frequency of the C=C stretching vibration is 1706 cm^{-1}. These data are available upon request. The structure will be deposited later and published with all related information (manuscript in preparation).

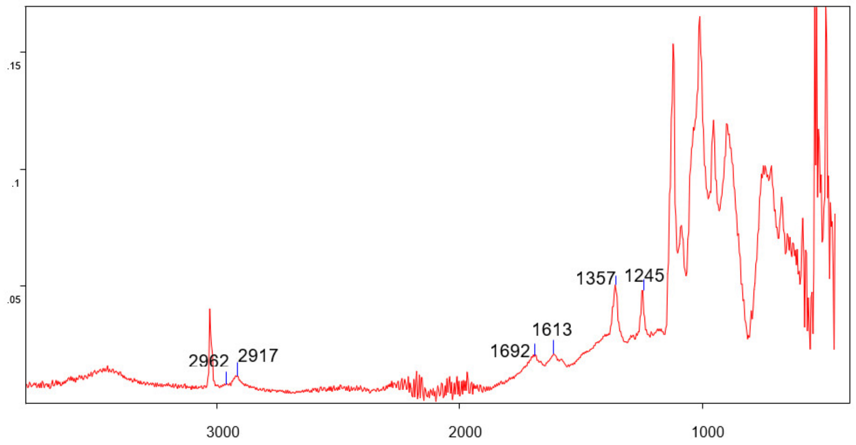

Figure S2. The ATR IR spectrum of proton disolvate **IVa**.

Table 1. Crystallographic data and details of the X-ray diffraction experiment.

Compound	Salt of cation I	Salt of cation IIa	Salt of cation III*
Empirical formula	$C_8H_{17}O_2 + B_{11}Cl_{11}CH$	$C_8H_{15}O + B_{11}Cl_{11}CH$	$C_6H_{13}O_2 + B_{11}Cl_{11}CH$
Formula weight	667.09 (663.9)	649.12	639.04
Temperature K	200(2)	200(2)	200(2)
Wavelength Å	0.71073	0.71073	0.71073
Crystal system	Monoclinic	Monoclinic	Monoclinic
Space group	$P2_1/n$	C2/c	$P2_1/n$
Unit cell dimensions *a* Å	10.8543(3)	34.904(3)	9.2079(5)
b Å	43.4962(11)	10.2295(9)	18.1850(11)
c Å	12.8025(3)	18.1986(16)	15.6536(10)
α °	90	90	90
β °	113.9110(10)	120.156(5)	93.992(2)
γ °	90	90	90
Volume $Å^3$	5525.6(2)	5618.5(9)	2614.8(3)
Z	8	8	4
Density (calcd.) $Mg.m^{-3}$	1.604	1.485	1.623
Abs. coefficient mm^{-1}	1.116	1.090	1.176
F(000)	2640	2464	1256
Crystal size mm^3	0.04 x 0.15 x 0.40	0.01 x 0.30 x 0.40	0.04 x 0.15 x 0.90
Θ range for data collection °	0.9 – 25.0	2.1 - 25.1	2.2 – 30.0
Index ranges	$-12 \leq h \leq 12$, $-51 \leq k \leq 51$, $-15 \leq l \leq 15$	$-41 \leq h \leq 41$, $-12 \leq k \leq 12$, $-21 \leq l \leq 21$	$-13 \leq h \leq 10$, $-25 \leq k \leq 25$, $-22 \leq l \leq 21$
Reflections collected	74208	46221	44516
Independent reflections	9746 R(int) = 0.066	4992 R(int)= 0.095	7137 R(int)= 0.042
Completeness to θ %	99.9	99.3	99.9 ($\theta \leq 50^o$)
Data / restraints / parameters	9746 / 4 / 616	4992 / 0 / 283	7137/ 0 / 291
Goodness-of-fit on F^2	1.08	1.02	1.08
Final R indices $I > 2\sigma(I)$	R_1= 0.0459, wR_2 = 0.1135	R_1=0.0486, wR_2 = 0.1102	R_1=0.0366, wR_2 = 0.0876
Final R indices (all data)	R_1= 0.0577, wR_2 = 0.1207	R_1=0.0898, wR_2 = 0.1298	R_1=0.0569, wR_2 = 0.1066
Largest diff. peak / hole $e.Å^{-3}$	0.72/ -0.59	0.80 / -0.70	0.82/ -0.35
CCDC			

Compound	Salt of cation IVa	Salt of cation IVb
Empirical formula	$C_6H_{13}O_2 + B_{11}Cl_{11}CH$	$C_3H_7O + B_{11}Cl_{11}CH$
Formula weight	638.03	580.96
Temperature K	200(2)	200(2)
Wavelength Å	0.71073	0.71073
Crystal system	Triclinic	Monoclinic
Space group	P-1	$P2_1/c$
Unit cell dimensions *a* Å	9.2804(10)	10.330(3)
b Å	11.6046(11)	13.184(3)
c Å	13.6551(15)	17.286(5)
α °	90.505(4)	90
β °	108.338(4)	92.933(9)
γ °	109.796(4)	90
Volume $Å^3$	1302.4(2)	2351.1(11)
Z	2	4
Density (calcd.) $Mg.m^{-3}$	1.627	1.641
Abs. coefficient mm^{-1}	1.180	1.295
F(000)	626	1128

Crystal size mm^3	0.10 x 0.20 x 0.20	0.08 x 0.10 x 0.55
Θ range for data collection °	1.6 – 27.6	1.9 – 25.0
Index ranges	-12 ≤ h ≤ 12, -15 ≤ k ≤ 14, -17 ≤ l ≤ 16	-12 ≤ h ≤ 12, -15 ≤ k ≤ 15, -20 ≤ l ≤ 20
Reflections collected	26608	30701
Independent reflections	5819 R(int) = 0.045	4158 R(int) = 0.149
Completeness to θ %	99.6	99.9
Data / restraints / parameters	5819 / 0 / 287	4158 / 186 / 282
Goodness-of-fit on F^2	1.02	1.01
Final R indices $I > 2\sigma(I)$	R_1=0.0542, wR_2 = 0.1406	R_1 = 0.1997, wR_2 = 0.4534
Final R indices (all data)	R_1=0.0839, wR_2 = 0.1627	R_1 = 0.2482, wR_2 = 0.4765
Largest diff. peak / hole e.$Å^{-3}$	0.97 / -0.51	2.47 / -1.03
CCDC		

* The reason for the poor R-factor is probably the disorder of the anion, which could not be localized.
